# Insights into the rehabilitation journey – patients’ experiences following tibial plateau fractures

**DOI:** 10.1080/17482631.2026.2636758

**Published:** 2026-02-25

**Authors:** Anna Fändriks, Michael Möller, Roy Tranberg, Annette Erichsen

**Affiliations:** a Department of Orthopaedics, Institute of Clinical Sciences, Sahlgrenska Academy, University of Gothenburg, Gothenburg, Sweden; b Institute of Health and Care Sciences, Sahlgrenska Academy, University of Gothenburg, Gothenburg, Sweden

**Keywords:** Knee injury, patient perspective, qualitative content analysis, qualitative method, tibial plateau fracture

## Abstract

**Purpose:**

The present study aimed to explore working-age patients' experiences with healthcare and recovery within the first two years after sustaining a tibial plateau fracture. By examining these perspectives, the study seeks to provide insights that can optimize rehabilitation strategies.

**Methods:**

Patients who had undergone surgical treatment for tibial plateau fractures were recruited. A qualitative descriptive design was employed, using semi-structured interviews analysed through inductive content analysis.

**Results:**

Nineteen patients from three hospitals in Sweden were interviewed 18–25 months after injury. Two overarching themes emerged: (1) *Journey to recovery*, with three categories on independence to adaptation, steps towards normality, and reshaping everyday life; and (2) *See me—guide me*, with two categories on the impact of information availability and interactions with healthcare professionals.

**Conclusion:**

The profound physical and psychological impact of tibial plateau fractures is underscored by the lengthy recovery and adaptation process. This journey is marked by significant challenges, but also by the resilience and resourcefulness of patients and their support networks. Interactions with healthcare services often introduce additional challenges, contributing to feelings of uncertainty and insecurity during critical times when patients require substantial support. Open communication, friendly attitudes, and clear action plans from healthcare professionals significantly enhance patient satisfaction and provide a sense of security.

## Introduction

Tibial plateau fractures—intra-articular fractures of the knee joint—have been shown to significantly impact patients’ lives, leading to persistent pain, mobility limitations, and other functional impairments over an extended period of time after injury (Prat-Fabregat & Camacho-Carrasco, 2016). These fractures affect individuals across all age groups, although they are generally observed in two primary patient populations: young adults after high-energy trauma and older adults after low-energy trauma (Prat-Fabregat & Camacho-Carrasco, 2016; Rudran et al., 2020). Simple falls, falls from significant heights, and traffic-related accidents are the most common mechanisms of injury (Albuquerque et al., 2013; Rozell et al., 2016; Wennergren et al., 2018).

Treatment modalities for these patients vary, encompassing both surgical and non-surgical approaches, depending on the fracture type and severity of the injury (Prat-Fabregat & Camacho-Carrasco, 2016). The most frequently employed surgical technique is open reduction and internal fixation (ORIF), typically involving the use of plates (Biz et al., 2019). Outcomes after a tibial plateau fracture range widely, from substantial deviations from pre-injury functional levels to excellent recovery. Persistent knee pain and instability are two common symptoms that continue to impede function, despite radiological evidence and range of motion indicating satisfactory healing (Ebrahimzadeh et al., 2015; Giannotti et al., 2016; Jansen et al., 2013; Raj et al., 2021). The younger, working-age patient population often experiences significant disruptions to daily life, particularly during the first six months post-injury, including work absenteeism and an inability to participate in previous sporting activities (Kugelman et al., 2017; O'Neill et al., 2024; Robertson et al., 2017; van Dreumel et al., 2015). Assessments of patient-reported Knee Injury and Osteoarthritis Outcome Score (KOOS) (Roos & Lohmander, 2003) outcomes have indicated reductions in the Quality of Life (QOL) and Sports Recreation subscales up to six years post-injury (Assink et al., 2024; Dobelle et al., 2024; O'Neill et al., 2024). Over time, reoperations for removal of fixation hardware are common (Wennergren et al., 2021). The development of knee osteoarthritis is another well-documented phenomenon in this patient population (Belluzzi et al., 2019), which, together with failed osteosynthesis, may ultimately necessitate total knee arthroplasty (Haslhofer et al., 2023).

Qualitative studies involving patients with other fractures in the lower extremities have highlighted that these injuries are associated with significant life impacts across physiological, physical, daily living, social and occupational, and domestic domains (McPhail et al., 2012). They also lead to long-term disruptions, including the process of feeling disempowered and changed, before adapting to a new self (Rees et al., 2019). However, there is a notable lack of studies focusing on patients' experiences and narratives in terms of the recovery process after tibial plateau fractures.

This study addresses a critical knowledge gap regarding rehabilitation experiences among working-age adults following tibial plateau fractures, exploring participants’ experiences using an interpretive descriptive approach. The primary objectives were to identify factors influencing physical and emotional well-being and to inform strategies for optimising treatment plans during the first two years post-injury.

## Materials and methods

This study employed a qualitative design with an interpretive descriptive approach (Thorne, 2025), focusing on participants’ subjective perspectives, understanding, and experiences of psychosocial wellbeing, care, and support. Sally Thorne’s interpretive descriptive design (Thorne, 2025) was employed because it is grounded in the aim of generating practical and clinically relevant insights. This approach emphasises addressing the central question, *“What does this mean for practice?”*, thereby ensuring that the findings are directly applicable to clinical contexts.

### Study population and procedure

Participants were recruited from three hospitals in Sweden—one university hospital and two regional hospitals—all of which follow similar routines for emergency and inpatient care. Upon registration at the hospital, fractures are classified according to the AO Foundation/Orthopaedic Trauma Association (AO/OTA) Fracture and Dislocation Classification Compendium (2007) (Marsh et al., 2007). Patients treated surgically typically remain on the ward for several days to allow clinical stabilisation. Early physiotherapy includes basic instructions for movement exercises and provision of walking aids, followed by individualised outpatient physiotherapy and orthopaedic follow-up, which usually involves radiographic evaluation.

**Figure 1.**
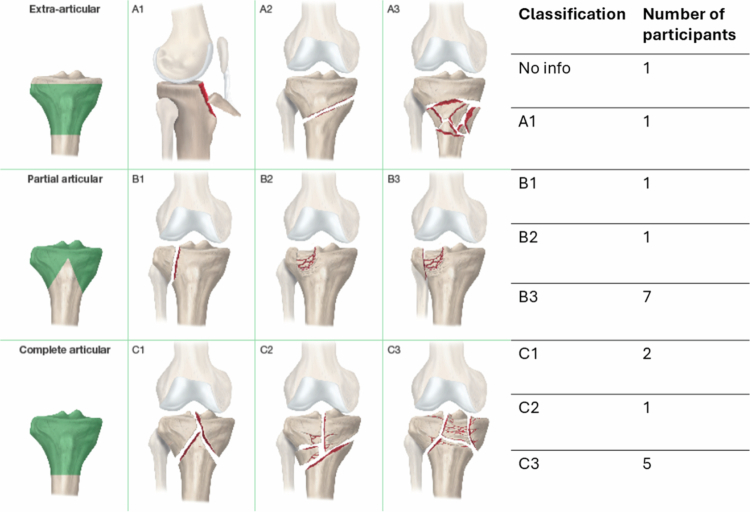
Schematic diagram of fracture classification according to the AO Foundation/Orthopaedic Trauma Association (AO/OTA) Fracture and Dislocation Classification Compendium (2007), along with the number of participants in each category.

The inclusion criteria are presented in Table I. Only individuals with surgically treated B- and C-type fractures were included (Marsh et al., 2007); these are intra-articular fractures of the tibial plateau with varying degrees of joint surface involvement (Figure 1). The participants of the current study were taking part in another study on gait function after a tibial plateau fracture. Reflecting the inclusion criteria of the gait function study, only those who could walk and had no other issues affecting their walking ability before the injury were included. Participants were recruited using purposive sampling to ensure diversity in age, gender, and hospital site. To maintain continuity in data collection, convenience sampling was also applied, and additional participants with similar characteristics (age, gender, and hospital site) were included, reflecting the limited size of the patient population.

**Table I.** Inclusion criteria for participation in the study.

Inclusion criteria
Unilateral, surgically treated B- or C-type tibial plateau fractures
No prior issues with gait function or other disabilities in the lower limbs
No previous neurological disorders
Ability to understand and communicate in Swedish
No cognitive disorders

Individuals who met the inclusion criteria were contacted by telephone 18-24 months after the index injury to enquire about participation in the study. Detailed information about the study was provided both orally and in writing, emphasising voluntary participation and the right to withdraw at any time. Interviews were conducted according to participant preference, either in person, by telephone, or via video meetings. Recruitment continued until data saturation was achieved, defined as the point at which no new codes or subthemes emerged (Younas et al., 2025). In practice, saturation appeared to occur after 17 participants had been interviewed. After a detailed discussion between the first and fourth authors, the decision was made to include two additional individuals to ensure data saturation.

### Data collection

Semi-structured interviews were employed. The first author (MSc, PhD student, female) created an interview guide with questions aimed to examine pre-defined subjects and to explore new perspectives (Appendix 1). The interview guide was reviewed and critiqued by the fourth author and an independent, experienced researcher in qualitative research, and subsequently by the second and third authors. The interview guide was structured around three main domains: (1) the injury, (2) the rehabilitation process and contact with healthcare professionals, and (3) the impact on daily life.

All interviews were conducted by the first author between September 2022 and September 2023 at a median of 21 months (range 18–25 months) after the injury. Two interviews took place in person in a neutral room in the hospital library, one was conducted as a video meeting, and the remaining sixteen were conducted over the telephone. Open-ended questions were used to provide in-depth and detailed data, in addition to the questions in the interview guide. Follow-up questions and prompts, such as “Tell me more about it” and “Can you clarify?” were incorporated during the interviews. All interviews were audio recorded and subsequently pseudonymized. The interview duration ranged from 18 to 70 minutes, with an average duration of 39 minutes.

The first author transcribed 15 of the 19 interviews. The remaining four were transcribed verbatim by a third party. The pseudonymized, transcribed data were securely stored in the University of Gothenburg’s servers, with access restricted solely to the first and third authors to prevent unauthorised access.

### Data analysis

Data were analysed using qualitative content analysis as described by Graneheim and Lundman (Graneheim & Lundman, 2004; Lindgren et al., 2020), utilising an inductive approach. The analysis was initiated at a manifest level, focusing on explicit content in the texts and identifying recurring patterns and expressions. Interviews were read and re-read to gain a sense of the whole by the first and fourth authors. In accordance with the study aim, the first author extracted meaning units from the interviews. Together with the fourth author, the meaning units were condensed while retaining their core essence. The data analysis proceeded with labelling the meaning units with codes, closely associated with the text. The first author grouped the codes based on similarities in their meaning or content, forming subcategories. These subcategories were summarised and further organised into broader categories that remained close to the text but included interpretative elements. The contents and titles within both subcategories and categories underwent discussion among all authors until consensus was reached, leading to progressive evolution as meaning units were moved between them. During this process, the number of subcategories and categories was gradually reduced. However, the original data was consistently reflected in the structure of both subcategories and categories (Table II). All coding was performed manually. After the data had been coded and categorised, the authors examined the patterns and relationships between categories, and a thematic layer was developed as the most abstract level, aiming to capture deeper meanings and latent dimensions of participants’ narratives. This process entailed searching for connections and contradictions within the data. Ultimately, two overarching themes were formulated that represented participants’ experiences on a broader, existential level.

Study participants did not provide feedback on the findings.

**Table II.** Coding tree illustrating the hierarchical structure of codes, categories, and themes developed during the qualitative analysis.

Statement	Code	Subcategory	Category	Theme
2: It has been quite tough. It really has. In the beginning, it was basically awful *laughs* because it was such an incredibly hard time at first, lying in the hospital for so many weeks and then all the pain.	Hospital stay and pain were difficult in the beginning	Navigating post-injury challenges and adaptations	From independence to adaptation: Overcoming post-injury hurdles	Journey to recovery
16: I wasn’t allowed to put any weight on the leg for three months. That was tough.	Difficult not being allowed to walk on the leg			
18: The first thing I did was go and buy an office chair. Otherwise, it was so hard to transport things while holding the crutches—it just didn’t work. So that was great.	Practical adaptation—bought an office chair instead of a wheelchair			
3: My family has been incredibly supportive.	Strong support from relatives	The crucial roles of relatives	From independence to adaptation: Overcoming post-injury hurdles	Journey to recovery
19: My partner could have helped me too, but it’s better that she works. So my mom and dad helped me instead and drove me to training.	Relatives drive			
2: I think that’s a huge challenge—learning to talk to patients in a way they understand.	Communicate so patients understand	The importance of being respected and involved in one’s own care	The impact of meeting and interacting with healthcare professionals	See me—Guide me
6: It was a nursing assistant… She was out of her mind. She hurt me, honestly. So I spoke to a doctor on the ward and said she wasn’t allowed to come in to me anymore.	Staff doesn’t listen to patient’s needs—causes harm			
7: They [the healthcare staff] have been absolutely fantastic… They’ve been very patient, very kind in showing understanding. Showing that they are there for me and that I’m not a burden.	Staff sees the patient—kind and shows understanding			
9: I think they [the medical clinic] should have contacted me earlier. Maybe after three months. Just to check that everything was okay. But they didn’t have time for things like that. I mean, I called several times and sent messages, but they never had time.	Hard to get follow-up appointments Deprioritized			
2: It was a very special situation when you couldn’t meet relatives and were admitted for quite a few months, or at least many weeks. Just feeling that support from the staff—that’s what made you feel good while you were there, I think.	Staff listens to needs and sees the patient—understanding, care, support, and commitment Alone during COVID—couldn’t meet relatives	The significance of support from and trust in healthcare personnel	The impact of meeting and interacting with healthcare professionals	See me—Guide me
6: Say after four or five days at the hospital, I felt pretty bad mentally. But then there was a nurse there, and I think she noticed that. Yes, she spent a lot of time talking.	Staff listens to needs and sees the patient—took time to talk Conversation support during hospital stay			

To ensure ***trustworthiness*** of the findings, as outlined by Lincoln and Guba and summarised by Bengtsson (2016), several methodological considerations were implemented throughout study planning, data collection, and analysis. To enhance ***credibility***, the study employed investigator triangulation, including discussions and consensus-building on the analysis process among several authors with diverse backgrounds related to the patient group and research. The interviews were conducted by the first author, an experienced physiotherapist, but a less experienced researcher. The first author was not involved in the clinical treatment of the participants. The second author is a senior fracture surgeon and an experienced researcher. The third author has experience in orthopaedic research but had more limited exposure to this patient group. The fourth author is a senior operating room nurse and researcher with expertise in qualitative research methods. The diversity in the professions and experience levels within the research group facilitated the capture of several perspectives. Recognising that the interviewer’s professional background could have influenced participant responses, ***reflexivity*** was addressed through regular peer debriefing and reflexive journaling to mitigate potential bias. Notes and flowcharts documenting the analytical process were maintained from the development of the interview guide through data collection and analysis, allowing for ***transparency*** of the study process. ***Confirmability*** was supported by systematic documentation of analytic decisions and the use of reflexive practices, ensuring that findings were grounded in the data rather than researcher bias. Tracking coding decisions and documentation of re-coding and relabelling were components of ensuring the ***dependability*** of the study. To enhance ***transferability***, participants of varied ages, equal representation of genders, and from three different hospitals were included in the study. To ensure accuracy, a professional third party translated the participant quotes from Swedish to English. Finally, this manuscript includes detailed descriptions of the data collection methods and the analytical process, enabling readers to evaluate the trustworthiness of the findings.

### Ethics

Participants were informed about the study's purpose, process, their ethical rights, and their ability to withdraw at any time. Information was provided twice: initially during the first contact in terms of the study participation and again at the beginning of each interview. All participants provided informed verbal consent, which was audio-recorded at the start of each interview. A subset of participants also submitted written informed consent. The intention was for all participants to provide written informed consent. However, some of those who participated digitally did not return signed consent forms, and in those cases, verbal consent was deemed sufficient. The content of both the verbal and written consent procedures was identical. Through either form of consent, participants confirmed that they had received both oral and written information about the study, had been given the opportunity to ask questions, and agreed to the handling of their personal data in accordance with the study protocol. To safeguard confidentiality, participants’ identities were replaced with identification numbers, and the data were stored on a university server accessible only to the researchers. In accordance with national legislation, ethical approval for the study was obtained from the Swedish Ethical Review Authority (registration number 2020-05869), with institutional affiliation to Department 2, Medicine.

## Results

Nineteen participants with surgically treated tibial plateau fractures were included in the study. The group comprised eleven women and eight men, with an average age of 51 years (range 29–66). All participants, except two, had an American Society of Anesthesiologists (ASA) Physical Status (PS) classification of 1–2. One participant was classified as ASA PS 3-4, and the classification was missing for one participant. The AO/OTA fracture classification breakdown is presented in Figure 1. Data for fracture classification were missing for one recruited participant and one participant’s fracture was reclassified from a B-type to an A-type after the data analysis.

The quantity and type of rehabilitation interventions varied among participants and were not accounted for in the study results. Information regarding potential associations with gender, age, treating hospital, or injury severity was intentionally omitted to safeguard participant confidentiality and prevent the risk of identification. At the time of the interviews, four participants had undergone reoperation to remove fixation materials. One of these participants had also undergone reoperation due to an infection and had been mobilised under anaesthesia due to joint stiffness. This participant was awaiting another mobilisation procedure, while three other participants had been scheduled to undergo or were considering knee arthroplasty in the future.

Data were compiled into 12 subcategories, abstracted into five categories, and finally consolidated into two overarching themes: (1) *Journey to recovery* and (2) *See me* **–** *Guide me* (Table III), with several subcategories.

**Table III.** Themes (1 and 2), categories (1–5), and subcategories that emerged from semi-structured interviews with patients following a tibial plateau fracture.

Theme 1: Journey to recovery
**1. From independence to adaptation: Overcoming post-injury hurdles**
Navigating post-injury challenges and adaptations
The crucial roles of relatives
**2. Steps towards normality: Triumphs and trials in rehabilitation**
Perceptions of recovery and milestones in rehabilitation
Balancing motivation and challenges in rehabilitation training
**3. Reshaping everyday life: Adaptation and development after injury**
Life after injury—Adapting to physical limitations
Psychological impact — Fears and altered mindsets
Future health concerns—The journey continues
**Theme 2: See me—Guide me**
**4. The impact of information availability on patients**
An informed patient manages better
Inadequate medical information negatively affects rehabilitation and employment
**5. The impact of meeting and interacting with healthcare professionals**
The importance of being respected and involved in one’s own care
The significance of support from and trust in healthcare personnel
The need for enhanced psychological support

### Theme 1—Journey to recovery

The theme *Journey to recovery* highlights the transformative journey patients underwent from the moment of injury onwards. The abrupt shift from independence to dependence had profound effects on mood and well-being, making the initial phase of recovery particularly challenging. Rehabilitation training and engagement with physiotherapists were essential for facilitating a return to normality, yet physical limitations and changes in participants’ mindsets persisted over time.

Theme 1 comprises three categories: *From independence to adaptation: Overcoming post-injury hurdles, Steps towards normality: Triumphs and trials in rehabilitation,* and *Reshaping everyday life: Adaptation and development after injury.*

### Category 1—From independence to adaptation: Overcoming post-injury hurdles

#### Navigating post-injury challenges and adaptations

Most participants found the post-injury period to be challenging, as they transitioned from independence to reliance on mobility aids and assistance from others. The inability to walk was seen as a significant barrier to daily activities, work, and leisure, and led to feelings of limitation and helplessness. Despite limited guidance from healthcare professionals, participants implemented their own solutions, including the use of shower chairs and sport braces, rearranging furniture, using an office chair when a wheelchair was not available, and switching cars for easier access.

*Life became extremely limited. I remember how long it took me to get up on the second floor to the living room—oh my god!* (Participant 3, female)

*From the beginning, I depended on others, including my husband, and could not care for myself. For three months, I could barely walk, and I felt helpless and abandoned. Yes, I was simply dependent. Maybe it's also that I don't want to depend on anyone else, but… That was what bothered me the most.* (Participant 7, female)

Most participants took a period of sick leave due to their injury. While some valued the time off, others found it tedious due to physical limitations or felt sorrow for not being able to work. A part-time return to work was beneficial for some, but for others, it was stressful and unrewarding. Two participants were forced to quit their previous jobs due to the injury. Effective communication with managers, including task adjustments, was key to successful returns to work.

*I can't work. I was sad; I was depressed. Then I didn't feel well.* (Participant 14, male)

*My boss arranged for me to come back early. I worked two hours a day for a few months. Now, I work 100%, but that's thanks to the fact that I have the job I have. It wouldn't have worked if I had a heavier job, one as physically demanding as before.* (Participant 6, male)

*Yes, but the worst began afterwards with the employment service and everything else. First, I would be on sick leave. "No, you shouldn't be on sick leave because you're healthy." "What's healthy? I can't take the kind of jobs I've had before." ...//... It's been hell.* (Participant 17, male)

#### The crucial roles of relatives

Relatives had crucial roles in supporting the participants’ post-injury recovery process, providing emotional and practical support. Although the help was appreciated, dependency on others led to feelings of vulnerability and guilt, as well as disrupted personal life plans, causing grief over shattered future aspirations. Relationships with relatives were also tested due to mood swings or the inability to engage in shared activities due to the injury.

*So, she [his wife] did a tremendous job when I got home. She took a month off. I couldn't get into the shower. Nothing. We had five steps up to the shower. She has done a tremendous job. Family is very important. …//… If I hadn't had my wife and two children when I got home, I probably would have gone under. I am absolutely convinced of that.* (Participant 6, male)

*My partner has been a huge help, so it's all good. But there was a time, as I said, when I wasn’t feeling so good, and it also affected my partner.* (Participant 14, male)

### Category 2—Steps towards normality: Triumphs and trials in rehabilitation

#### Perceptions of recovery and milestones in rehabilitation

Participants perceived the overall recovery period as long and occasionally slow-going. Some expressed frustration due to the lack of information regarding the duration of rehabilitation, while others were content with not knowing the exact length of the process in advance. The point at which individuals felt they had returned to a more normal life varied, but engaging in increased physical activities, such as walking, mowing the lawn, or climbing stairs, was considered a significant marker of normality. For some, this turning point was perceived to occur approximately one year post-injury.

*Almost a year later, I think I felt like it was starting to be an everyday grind again, and maybe it had already been like that before as well, but I personally felt I was a little more in place, like how it was before.* (Participant 10, female)

#### Balancing motivation and challenges in rehabilitation training

Rehabilitation training was considered essential for well-being and recovery. Even the participants who had not engaged extensively in training emphasised its importance and expressed regret for not doing more. However, resistance to training was noted, often due to perceptions of it being dull and uncomfortable. Additionally, doubts about benefits and distractions at home contributed to participants forgoing rehabilitation exercises.

Progress was identified as the primary motivator to continue, alongside the desire to resume previous activities to avoid long-term dependency on others. Many participants observed that overtraining periodically hindered progress, highlighting the importance of maintaining a balanced exercise intensity to prevent setbacks.

It's kind of up and down… Then so, “Now, damn” (*laughs) I train like crazy, and sometimes it gets too much. Then there are setbacks, so I've learned to try to keep this steady pace, according to her [physiotherapist’s] instructions, so then it works great, and it goes forward even if it's incredibly slow. That was not the time perspective I had in mind. (Participant 3, female)

*Do the rehab; you win from it. Also, how damn boring it is. …//… The strategy, then, is that you may want to feel better and be a more mobile person again. Thus, you want to live and enjoy your life without being limited.* (Participant 19, male)

### Category 3—Reshaping everyday life: Adaptation and development after injury

#### Life after injury—Adapting to physical limitations

Many participants continued to experience incomplete recovery at the time of the interviews, recognising the potential lifelong impact of their injuries. Returning to previous activity levels was challenging despite intensive rehabilitation efforts. Persistent issues often dominated daily thoughts, with the affected knee constantly reminding participants of its presence.

*I can move. But I feel this leg all the time, I can say that.* (Participant 12, female)

And the only thing I can think about is that I can't walk, I can't drive my car, I can't do anything... (*raises voice) I can't do anything, I can't do anything. (Participant 3, female)

Pain was a primary limitation, affecting daily activities and sleep. Participants reported localised knee pain or pain radiating throughout the leg, with stair climbing and walking frequently triggering discomfort. Compensatory movements led to pain in other body areas. Reduced knee mobility was common, and some participants had surgeries to address mobility issues. Others experienced swelling after activities or stiffness.

*I always have an intense feeling in my leg. It's most noticeable when I go up and down the stairs and take long walks, like getting on and off streetcars and buses. You know, there are obstacles on the ground; I can't run. That alone is a dramatic difference. I can't run to the streetcar. It hurts.* (Participant 2, female)

These issues led to significant lifestyle changes. Some participants avoided activities to prevent pain or due to a lack of trust in their knee, while others stopped activities altogether. Some voluntarily changed their routines, while others felt forced to abstain from activities they once enjoyed, leading to feelings of sadness and frustration, such as missing out on children’s activities or family outings.

*I can't do things how I want at home—that is not possible. I can't go into the woods. Walking on uneven ground hurts a lot, and I love the forest.* (Participant 6, male)

#### Psychological impact — Fears and altered mindsets

Several participants experienced psychological effects following injury, questioning whether their discomfort was physical or psychological. Fear of reinjury or falling led to distrust in their knee, cautiousness, and avoidance of certain activities, resulting in increased isolation and anxiety for some. However, some found a positive shift in mindset, focusing more on the present. A number of participants recalled being informed by doctors during follow-up appointments that their knee had healed, and that they could resume demanding activities like skiing or hiking if they felt ready. Despite this reassurance, several participants remained hesitant, fearing re-injury.

*I think it has changed because I have inner concerns. …//… And that I'm more careful. Even if I try to challenge myself.* (Participant 12, female)

*I'm afraid of falling, I can say, for example. I'm terrified of falling again. …//… It's in my head; that's just how it is. You don't want it to happen again, so that's why you do that.* (Participant 18, female)

Despite ongoing discomfort and challenges, many adapted to their issues and perceived their functionality as good relative to the severity of the injury. Some expressed gratitude for what they were still able to do, such as being able to walk.

#### Future health concerns—The journey continues.

Several participants emphasised the importance of continuing training for future health, maintaining physical ability, and preventing future issues. Some faced upcoming evaluations and surgeries, which evoked a mix of hope and fear. While some hoped for gradual improvement and acknowledged their adaptation to knee function, others worried about long-term consequences, wondering how it might impact them at an advanced age.

*He showed me what a normal prosthetic change is like, but this is much worse, he said. So I'm a little scared it won't be as good as I would like, but I hope so.* (Participant 6, male)

*No, but I'm afraid that... It will hurt a lot when I reach my 60 s or 70 s, and it will be worse. This is a big fear of mine.* (Participant 8, female)

### Theme 2: See me—Guide me

The theme *See me—Guide me* refers to the participants' desire for increased assistance and guidance from healthcare personnel during the recovery process. The importance of being informed and involved, regardless of background, was emphasised. Access to information and the way staff interacted with patients were two critical factors that permeated the entire recovery process from the time of injury to the time of the interview. Theme 2 comprises two categories: *The impact of information availability on patients* and *The impact of meeting and interacting with healthcare professionals.*

### Category 4—The impact of information availability on patients

#### An informed patient manages better

At the emergency department, participants experienced long waiting times without information regarding treatment plans. Coupled with minimal assistance from healthcare personnel, the situation left participants feeling anxious, uncertain, abandoned, and uninformed.

*It felt like I waited forever; you got stuck in the emergency intake site the whole time. You joked and said, "It's not the healthcare that's the issue; it's the waiting time."* (Participant 5, male)

*And then, in the emergency room, I had to lie there and wait a long time. Nobody cared about me. I was in a lot of pain. No one checked, offered pain relief, or anything. …//…* *Did I get anything to eat? No. I just lay there all alone and couldn't do anything. It was painful. So really, really damn painful.* (Participant 12, female)

Surgery waiting times were also perceived as long. Participants who were sent home while awaiting surgery expressed a desire for more continuous information to alleviate anxiety. Conversely, those who remained in the hospital while awaiting surgery felt safe and well-informed during their hospital stay.

#### Inadequate medical information negatively affects rehabilitation and employment

Several participants stated that they received inadequate information about their injury and surgery throughout the recovery period. Contributing factors included difficulties in understanding medical information and challenges in scheduling follow-up appointments, which hindered opportunities to discuss their injury. This lack of information delayed some participants' comprehension of their injury and negatively impacted their rehabilitation, leading to a focus on incorrect aspects. The absence or inadequacy of medical information also impacted sick leave and job application processes.

*Be clearer about what has happened and what was done after the injury. This would have also helped me better with my rehab. How should we work with the rehab as well? They have focused completely on the wrong things because they have not been completely clear in their communication about what was done during the operation. …//… If I had known that and my physiotherapist had known it, it would have been different.* (Participant 2, female)

### Category 5—The impact of meeting and interacting with healthcare professionals

#### The importance of being respected and involved in one’s own care

Respect, attentive listening, and active involvement in care were pivotal for the participants. Many highlighted the exemplary services provided by nursing staff during their hospital stay, while others experienced that some healthcare professionals disregarded their individual needs, leading to discomfort, increased pain, and feelings of insecurity.

Clear communication and supportive language were crucial for reassurance, while negative attitudes, particularly from orthopaedic surgeons, made participants feel like a burden and hesitant to ask questions. According to participants, improvements were needed in terms of attitudes, language, and demonstrating willingness to assist patients. Those who reported positive experiences with their consultations with orthopaedic surgeons consistently praised the provision of clear information and the surgeons' openness to discussion.

*It was tough in the hospital, but the nurses were amazing. They always made sure to explain every little thing they were doing. I perceive it as very, very good and positive. Even if the incident was very difficult and negative, everything else has been great. Honestly, the care couldn't have been any better.* (Participant 16, female)

*He [the surgeon] had no time or desire to help me at all. …//… "You can adjust to the fact that you probably won't be able to work anymore. And you can expect to get a knee replacement". So overall, it was very negative news. …//… It could have been set up completely differently.* (Participant 11, male)

Several participants reported that their interactions with healthcare services were terminated prematurely. They noted a distinction between feeling personally "ready" and being deemed "ready" by the healthcare system, expressing a desire for long-term follow-up and support. Participants wished to follow up on both known issues and stagnating recovery processes. Doctors and physiotherapists who provided opportunities for ongoing questions or check-ups helped participants feel more at ease.

*As long as you still feel like you're struggling, as I mentioned …//… it would have been incredibly important for me to have such a follow-up. Because then you would feel like, 'Okay, they won't let go until you're better.* (Participant 2, female)

*I had contact with the orthopaedic surgeon for six months afterward, and since then, I haven't had it. Maybe it could have been better. But I made good progress in the beginning. I met the person who operated on me after six months. At that time, I had just stopped using the crutches, and then he said it had turned out well because he probably didn't think I would stop using the crutches even after six months. But then it didn't get any better.* (Participant 15, male)

#### The significance of support from and trust in healthcare personnel

Support from healthcare personnel was considered crucial during the initial hospital stay, when feelings of vulnerability and exposure were common. The prohibition of family visits during the COVID-19 pandemic made this support even more important. Post-discharge, physiotherapists were considered the primary source of support. Participants valued being provided with tailored information in terms of their injury, surgery, and rehabilitation training, as well as physical assessments, and motivating words.

*Yes, a fantastic physiotherapist who gave me support, training, and tips, and yes, they stopped me when I thought everything was going too slowly.* (Participant 7, female)

Some participants were dissatisfied with their initial physiotherapist and sought care from another provider. Reasons for dissatisfaction included a lack of personal connection or perceived competence. Generally, trust in healthcare professionals varied, with those demonstrating competence and willingness to learn being trusted more. Communication between healthcare professionals also influenced trust.

*Those who understood my injury treated me properly.* (Participant 6, male)

#### The need for enhanced psychological support in early recovery

Some participants highlighted the psychological challenges they faced, noting a lack of mental health support. They emphasised the need for improved psychological support, both early on and in the months following the injury, as the focus was mainly on their physical condition. Independently, some participants found help through workplace psychologists or discussions with their physiotherapist. Support from friends and family with similar experiences was also appreciated.

*They are so concentrated on the body that they forget what is also important, which is a huge part of rehabilitation.* (Participant 2, female)

*But I think you should be able to have someone to talk to from day one. Because I mean, it's a trauma.* (Participant 15, male)

#### Suggestions for improvements

Based on the reflections and experiences of the participants, as well as interpretations by the authors, the findings have been synthesised into a set of recommendations for improvements and considerations for healthcare professionals working with patients with tibial plateau fractures (Appendix 2).

## Discussion

The aim of this qualitative study was to explore working-age patients’ experiences of healthcare and recovery within the first two years after a tibial plateau fracture. The primary findings of this study confirm that recovery following orthopaedic trauma is multifaceted—extending beyond physical repair to include psychological and social dimensions. Two overarching themes emerged: *Journey to recovery* and *See me—Guide me*. The former captures participants' reflections on physical, psychological, and emotional transformations post-injury, while the latter underscores the profound impact of information and collaboration with healthcare professionals. In this discussion, selected aspects will be highlighted and interpreted in light of established literature and conceptual models.

The theme *Journey to recovery* was characterised by a significant transition from independence to dependence, making the initial phase after injury particularly challenging both practically and psychologically. In the category *From independence to adaptation: Overcoming post-injury hurdles*, participants described the challenges of acknowledging their inability to care for themselves and expressed feelings of guilt toward relatives who had to exert additional effort to support them. Similar experiences have been reported in patients with ankle, hip, and other lower extremity fractures, where dependency on others is frequently linked to disempowerment and emotional distress (McPhail et al., 2012; Rees et al., 2019; Tutton et al., 2018; Ziden et al., 2008, 2010). While none of our participants reported suicidal ideation, the narratives suggest that early dependency can exert a substantial impact on mental health and overall well-being, echoing prior work that situates loss of autonomy as a salient stressor in musculoskeletal trauma (Rees et al., 2019).

The categories *Steps towards normality: Triumphs and trials in rehabilitation* and *Reshaping everyday life: Adaptation and development after injury* reflected a gradual return to independence, albeit with enduring physical or psychological adaptations relative to the pre-injury state. Participants emphasised the salience of reclaiming prior functional skills and that perceived progress or setbacks influenced their mood, motivation, and engagement. These findings are consistent with previous orthopaedic studies that highlighted acceptance and return to work as pivotal components of recovery (McPhail et al., 2012; Rees et al., 2019; Tutton et al., 2018). Notably, earlier research has described processes that individuals engaged in, such as “recreating a new me”, integrating their pre-injury identities with present capabilities (Rees et al., 2019). In the present study, explicit identity reconstruction and acceptance were less prominent. However, participants described changes in activities, thought patterns, and social relationships—consistent with recovery as a complex process of adaptation.

The participants’ narratives align with the broader illness trajectory framework described by Halcomb and Davidson (2005), which conceptualises recovery as a pathway shaped not only by tissue healing but also by ongoing symptom monitoring, self-management, and the social consequences of injury for the individual and their loved ones (Halcomb & Davidson, 2005). In parallel, the integrated model of psychological response to sport injury described by Wiese-Bjornstal et al. foregrounds the interplay between cognitive appraisals, emotional responses, and behavioural strategies, modulated by personal and situational factors (Wiese-bjornstal et al., 1998). Although our cohort is not limited to athletes alone, the model offers a useful lens in interpreting the findings. In line with the model, positive thoughts about coping (constructive appraisals) appeared to increase participants’ motivation and commitment to their rehabilitation exercises, while negative thoughts reduced their engagement. The model also reminds us that recovery is not fixed; emotions and behaviours change over time with pain levels, physical function, social support, and how clear the information is (Wiese-bjornstal et al., 1998)—factors repeatedly highlighted in our interviews.

The second theme, *See me—Guide me,* reflects a consistent call for healthcare that is person-centred, respecting individual preferences, capacities, and context, and informationally robust, enabling patients to understand their injury and care plan (Coulter & Oldham, 2016; Ekman et al., 2011). Participants reported gaps at both interpersonal and organisational levels (e.g., variability in caregiver attributes, follow-up routines, and information delivery). These gaps were identified during the whole recovery process, but were particularly challenging, both psychologically and physically, in the acute phase of injury.

Consistent with prior work in emergency care settings, participants perceived the emergency department as a critical setting where communication quality, timeliness, and comprehensiveness directly shape feelings of safety, trust, and dignity (Milton et al., 2023). Caring approaches and effective dialogue were linked to reassurance. Conversely, scarce information about scheduling, waiting times, or care pathways fuelled uncertainty and left patients feeling forgotten and deprioritized (Milton et al., 2023).

Orthopaedic surgeons were characterised as a primary source of information and figures carrying significant responsibility. Participants emphasised that clear communication must be accompanied by an appropriate attitude to foster safety and trust. Samsson et al. (2017) showed that patients’ expectations before consultations are shaped by prior experiences of the surgeon’s demeanour, underscoring the importance of respectful interactions. In our interviews, participants who engaged in well-informed discussions reported feeling satisfied and secure, suggesting that content quality, relational tone, and shared decision-making are important factors for effective communication with healthcare professionals.

Participants in the present study specifically requested two informational domains: unit-specific treatment plans and injury-specific information. Providing written materials (e.g., Appendix 2) is a pragmatic strategy to standardise core facts, improve recall, and support self-management. Prior literature suggests that well-informed patients are more confident in managing their own care (Wolf et al., 2017), communicate their needs more effectively (Stenner et al., 2018), and make better-aligned decisions about rehabilitation when guidance is clear and timely (Ziden et al., 2010). These insights point toward operational improvements that are feasible to implement within current service structures.

In the subcategory *The significance of support from and trust in healthcare personnel*, physiotherapists were identified as a significant source of support for participants over time across both psychological and physical domains. Prior work in long-term recovery from open lower-limb fractures similarly highlights the role of physiotherapy in reducing uncertainty and structuring purposeful rehabilitation (Rees et al., 2019). Our participants’ narratives indicate opportunities to strengthen continuity of care and extend follow-up, including mid-term reviews with orthopaedic surgeons to evaluate healing progress and knee capacity for strenuous activities, complemented by thorough physical assessments by physiotherapists. For patients with persistent problems (e.g., ongoing pain), participation in pain management or osteoarthritis programmes may be beneficial (Wride & Bannigan, 2018). More broadly, personalised rehabilitation plans should be developed and periodically revised as conditions and priorities evolve, incorporating modified exercises and equipment tailored to individual needs and activity levels (Ekegren et al., 2020).

The subcategory *The need for enhanced psychological support* highlights requests for more support, particularly in the early stages post-injury. Guidelines for complex rehabilitation recommend early psychological and psychosocial interventions, including listening to patients' concerns to identify those with pre-existing psychological symptoms in need of specialised support. Guidelines also advise normalising common post-traumatic psychological reactions while remaining vigilant for cases requiring additional care (National Guideline Alliance, 2022). Enhanced psychological support could better address patients' feelings of uncertainty and issues with self-identification—recurrent elements in our data.

The two themes identified in this study not only complement each other but also interact in meaningful ways. The interplay between patients’ internal processes and their interactions with healthcare systems and professionals can be interpreted through both the Illness Trajectory Framework (Halcomb & Davidson, 2005) and the International Classification of Functioning, Disability and Health (ICF) framework developed by the World Health Organisation (2025). These frameworks emphasise the importance of considering individual characteristics alongside contextual factors that may serve as barriers or facilitators to recovery. While addressing the full spectrum of physical and psychological concerns remains a considerable challenge, adopting a long-term perspective on patient needs, combined with respectful engagement from all healthcare professionals and comprehensive information sharing, has the potential to improve and simplify several aspects of the recovery journey for patients with tibial plateau fractures.

Although qualitative data is not usually generalisable, we consider our findings to be transferable to other patients with tibial plateau fractures because of shared clinical constraints and rehabilitation requirements. Regarding experiences with healthcare professionals, we believe the narratives from this study can be applied to many patient groups, particularly within orthopaedics. In terms of walking ability and independence, the strict weight-bearing restrictions for this patient group—stricter than for many other lower limb injuries—may limit transferability to some extent. Nevertheless, walking difficulties and reliance on walking aids are also common in other lower limb injuries and, to some degree, in spinal conditions, making parts of these narratives relevant to a broader patient population. While our study focused on working-age individuals, the findings are largely applicable across a wider age range. However, fracture care is individualised, and not all observations will apply universally.

### Strengths and limitations

This study contributes nuanced, patient-centred accounts of recovery after tibial plateau fracture and situates the findings within established recovery models and person-centred care principles. However, several limitations warrant caution. The sample was recruited from a **single country**, which may restrict transferability to other contexts and healthcare systems. **Digital interviews** may have influenced the relation between participants and the interviewer, and the depth of data gathered may be more limited compared to in-person settings. **Member checking/validation was not conducted**, limiting opportunities to verify researchers’ interpretations with participants. Recall bias is also possible, particularly concerning early-phase experiences.

## Conclusion

The profound physical and psychological impact of tibial plateau fractures is underscored by the lengthy recovery and adaptation process. This journey is marked by significant challenges, but also by the resilience and resourcefulness of patients and their support networks. Interactions with healthcare services often introduce additional challenges, contributing to feelings of uncertainty and insecurity during critical times when patients require substantial support. Open communication, friendly attitudes, and clear action plans from healthcare professionals significantly enhance patient satisfaction and provide a sense of security. Integrating structured psychological support, maintaining consistent communication, and incorporating extended follow-ups have the potential to improve recovery outcomes and patient satisfaction after tibial plateau fractures.

## Data Availability

The data that support the findings of this study are available from the corresponding author upon reasonable request.

## Appendices

- How has the time since the injury until today been for you?
- How do you perceive the rehabilitation process that you have undergone?
- Could you describe your experience at the hospital?
- How do you feel about the support from healthcare professionals during this time?
  ∘ Can you mention something specific that has been good?
  ∘ Something that could have been done differently?
- Do you feel that your life has changed in any way after the knee injury?
  ∘ If yes: In what way?
  ∘ If no: What do you think has contributed to your successful recovery after the injury?
- What have you struggled with the most?
- Have you employed any strategies to facilitate rehabilitation after the injury?
- For example: When you describe (e.g., pain), what do you do?
- What has been important to you after this injury?
- How do you think about the future considering the injury you have experienced?
- If you were to meet someone who has had the same injury as you, what would you like to say to that person?

***Appendix 1.** Semi-structured interview guide.*

**Suggestions for improvements in care to offer effective treatment and support after a tibial plateau fracture.**

- Complement physical treatments and consultations with offers of professional psychological support both early in the recovery process and several months after the traumatic injury.
- Extend the follow-up period with the orthopaedic surgeon.
- Implement long-term follow-ups with assessments of physical function with a physiotherapist.
- Follow-ups with the orthopaedic surgeon and physiotherapist should ideally be synchronised.
- Consider offering participation in a pain management programme.
- Support and guide patients in adapted physical activities to facilitate physical exercise.
- Provide clear communication channels with healthcare professionals and conclude care in consultation with the patient.

**Factors for healthcare professionals to consider when meeting with patients with tibial plateau fractures.**

- Take time, listen, and provide clear explanations regarding the injury, restrictions, recommendations and the proposed rehabilitation plan.
- Communicate clearly and be mindful of how you convey information.
- Encourage the patient to understand their injury better and to know what assistance they can receive.
- Patients appreciate healthcare professionals who have “read up” on the patient's medical history and current injury.
- Patients appreciate healthcare professionals who are willing to learn more when they lack knowledge.
- Do not criticise other healthcare professionals in front of the patient regarding differences in views on medical information and treatment options.
- Be mindful of what you say in open corridors and treatment areas. Patients easily overhear and are influenced by it.
- As follow-up with the orthopaedic department comes to an end, the physiotherapist often becomes the main point of ongoing care and support.
- Provide tailored exercises and collaborate with the patient to identify their rehabilitation goals, thereby enhancing engagement in exercises that may otherwise feel less motivating.
- Encourage and affirm the patient to seek help from family members during the initial recovery period.

**Suggestions for written information to provide to the patient:**

Injury: Name of the injury.

Intervention: What was done during the surgery?

Plan: (e.g., follow-up appointments).

Rehabilitation timeline:

Restrictions and recommendations:

- Weight-bearing/off-loading:
- Compression stockings:
- Brace:

Contact person for questions:

Communication channels upon discharge:

***Appendix 2.** Suggested factors that may contribute to facilitate the rehabilitation process following a tibial plateau fracture*
